# Insights Into Sequences of Viral and Bacterial Origin in the Metatranscriptome of *Centaurea cyanus* L. Susceptible and Resistant to Acetolactate Synthase (ALS)‐Inhibiting Herbicides

**DOI:** 10.1111/1758-2229.70287

**Published:** 2026-02-05

**Authors:** Katarzyna Marcinkowska, Barbara Wrzesińska‐Krupa, Aleksandra Obrępalska‐Stęplowska

**Affiliations:** ^1^ Department of Weed Science and Plant Protection Techniques Institute of Plant Protection, National Research Institute Poznań Poland; ^2^ Department of Molecular Biology and Biotechnology Institute of Plant Protection, National Research Institute Poznań Poland

**Keywords:** ALS‐inhibitors, herbicide resistance, microbiome, transcriptome, virome, weeds

## Abstract

Cornflower ( *Centaurea cyanus* L.) is a widespread weed in cereal crops and is commonly controlled with sulfonylurea herbicides. In Poland, populations of cornflower resistant to acetolactate synthase inhibiting herbicides, such as tribenuron‐methyl, have been increasingly reported. Both target‐site and non‐target‐site resistance mechanisms may contribute to this phenomenon. Plant‐associated microorganisms are known to play essential roles in alleviating abiotic stress. Moreover, weeds are considered reservoirs of plant pathogenic viruses. Since bacteria and viruses associated with cornflower have not been analysed to date, data mining was undertaken to identify viral and bacterial sequences in metatranscriptome datasets obtained from plant biotypes that are both susceptible and highly resistant to tribenuron‐methyl. Using MEGAN6 and Kraken2, taxonomic classification revealed the presence of sequences of two double‐stranded RNA viruses belonging to the family *Partitiviridae*, which have not been described before. For bacterial sequences, 19 genera were identified, including *Bacillus*, *Mesorhizobium* and *Acinetobacter*, some of which are associated with plant growth promotion or xenobiotic degradation. Although the presence of partitiviruses was unrelated to herbicide resistance status, some bacterial genera (e.g., *Rothia*) were more abundant in resistant than in susceptible plants. These results suggest that those bacterial genera present in weeds may be involved in counteracting ALS‐inhibiting herbicides.

## Graphical abstract

Metatranscriptomic analysis of *Centaurea cyanus* revealed diverse plant‐associated viral and bacterial sequences in herbicide‐susceptible and ‐resistant biotypes. While novel partitiviruses occurred independently of resistance status, differences in bacterial community composition may suggest a potential role of the plant microbiome in responses to herbicide stress.

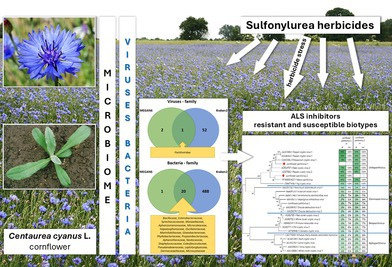

## 1 Introduction

Weeds infestation is a global challenge faced by the farmers for every field crop, despite considerable differences in the cropping systems. An attempt is made by the farmers to control these weeds as to protect current and future harvests (Nthebere, Tata, Bhimireddy, et al. 2025a; Nthebere, Tata, Gudapti, et al. 2025b; Ramprakash et al. 2024). One of the most important problems related to weed control in crops is the phenomenon of weed resistance to commonly applied herbicides. The first reports on this subject were published in the 1950s (Switzer 1957). The problem of weed resistance has intensified over time because of, among others, excessive herbicide use (Nthebere, Tata, Bhimireddy, et al. 2025a; Peterson et al. 2018). Today, more than 273 weed species all over the world have developed herbicide‐resistant biotypes (Heap 2025). Resistant weeds occur in 102 crops across 75 countries.

The long‐term use of herbicides, mainly those with one mode of action, results in the systematic elimination of susceptible individuals from the population. This creates conditions for the spread of resistant biotypes, which, after some time, become the dominant element in the population (Gaines et al. 2020; Peterson et al. 2018).

Currently, the resistance of dicotyledonous weeds is less important than that of monocotyledonous ones (Stankiewicz‐Kosyl et al. 2021). Nevertheless, reports of resistance of cornflower to acetolactate synthase (ALS) inhibitors, i.e., herbicides from group 2 according to the Herbicide Resistance Action Committee (HRAC), are becoming increasingly frequent (Stankiewicz‐Kosyl et al. 2024). In the last few decades, there has been a significant increase in the production of winter crops in Poland, especially wheat and oilseed rape, which makes winter weeds, such as cornflower, a greater threat to crops (Guillemin et al. 2022; Stankiewicz‐Kosyl et al. 2020; Wacławowicz et al. 2022). It is even more visible in the context of the emerging resistance of this species to herbicides. Cornflower is a very competitive weed against cultivated plants. One plant can produce approximately 700–1600 achenes, which fall out of the baskets just before the winter crops are harvested (Jursík et al. 2009; Petit et al. 2015). The economic threshold for the harmfulness of cornflowers in cereals is equal to 1–5 plants per metre (Wacławowicz et al. 2022). In recent years, a significant local reduction in the sensitivity of this species to herbicides has been observed (Stankiewicz‐Kosyl et al. 2020). Interestingly, so far, only in Poland, the occurrence of herbicide‐resistant cornflower has been confirmed. The resistance of cornflower to ALS inhibitors and herbicides from the synthetic auxin group (group 4 according to HRAC) was reported (Heap 2025). The first report from 2006 concerned the resistance of cornflowers to chlorsulfuron (Marczewska and Rola 2006), and the next report from 2010 indicated the progress of the problem of herbicide resistance because two tested biotypes were cross‐resistant to tribenuron methyl and chlorsulfuron (Adamczewski and Kierzek 2010).

Two herbicide resistance mechanisms have been defined. The first one, the target site resistance (TSR) mechanism, is associated with the mutations found at the action site of the enzyme, being the herbicide target or the increase in expression of the gene coding for the target enzyme (Powles and Yu 2010). The second mechanism, the non‐target‐site resistance (NTSR) mechanism, involves changes in the activity of several processes, such as decreased rates of herbicide uptake, penetration, activation, translocation or increased metabolism or sequestration of the herbicide (Powles and Yu 2010). Both TSR and NTSR mechanisms were suggested for the emergence of cornflower resistance to herbicides (Saja et al. 2016). Further studies aiming at the identification of the mutations contributing to TSR revealed mutations found at position P197 of the ALS amino acid sequence and several other mutations that have not been reported for their contribution to resistance emergence thus far (Stankiewicz‐Kosyl et al. 2024; Wrzesińska and Praczyk 2021). Importantly, the high‐expression genes coding for enzymes involved in detoxification processes, such as cytochrome P450 monooxygenases (CYP450s), glutathione S‐transferases, glycosyltransferases, ABC transporters, esterases and oxidases, are considered the main contributors to NTSR (Délye 2013). Moreover, the flowers of cornflower plants resistant to tribenuron‐methyl investigated in the context of their phytomedical properties exhibited significantly higher antioxidant activity, higher levels of total phenolic and flavonoid compounds, and higher reducing power than herbicide‐sensitive plants (Gawlik‐Dziki et al. 2023). The latter one additionally increased after herbicide treatment, which shows that herbicide resistance in this species might be related to changes in secondary metabolism, particularly in the accumulation of flavonoids. Those data suggest that the NTSR mechanism might also play a role in the herbicide resistance emergence in cornflower plants found in the Polish fields.

In a strategy to prevent the development of herbicide resistance, it is important to know which factors contribute to this phenomenon and the spread of resistant biotypes in the environment. Recent research increasingly shows that to the factors contributing to abiotic stress tolerance, including pesticide resistance/tolerance, belong host microbiome, especially host‐associated bacteria (Caddell et al. 2019; Govindasamy et al. 2018; Motamedi et al. 2022; Wielkopolan et al. 2024). The endophytes (Mei and Flinn 2010), including bacterial (Ryan et al. 2008) and fungal endophytes (Schardl et al. 2004), as well as viruses (Sabbadin et al. 2017; Xu et al. 2008), may also contribute to the increase of plant tolerance to abiotic factors. Some viruses have been shown to establish a beneficial relationship with their host in difficult environmental conditions (González et al. 2020). For example, tomato yellow leaf curl virus could make its host tolerant to very high temperatures and drought (Shteinberg et al. 2021).

Transcriptomic datasets are a good source to analyse the presence of microbial sequences in plants. Our previous analysis of the transcriptome of silky bentgrass plants resistant to pinoxaden (group 1 according to HRAC) showed that genes encoding detoxifying enzymes and those associated with plant–pathogen interaction pathways were highly expressed. Those data suggest that herbicide resistance status may also influence weeds' interactions with other stresses (not only abiotic but also biotic ones) or vice versa (Wrzesińska‐Krupa et al. 2023). Since cornflower was not analysed for the presence of the plant‐associated microbial communities thus far, the aim of this study was to analyse transcriptomic data for the presence of viral and bacterial sequences, both in plants belonging to susceptible and resistant populations of *C. cyanus* .

## 2 Materials and Methods

### 2.1 Greenhouse Experiments and Plant Material

The biological studies were carried out in a greenhouse under controlled environmental conditions: temperature 20°C ± 2°C, relative humidity 60% and a 16/8 h day/night photoperiod (Protocol S1). Plants were grown in 0.5 L pots filled with peat‐based potting substrate, thinned to five seedlings per pot within 10 days after emergence, and watered as required. Herbicide treatments were applied at the 2‐leaf stage (BBCH 12) using a moving‐nozzle sprayer delivering 200 L ha^−1^ through a TeeJet 1102 flat‐fan nozzle at 0.2 MPa, positioned 40 cm above the canopy and moved at 3.1 m s^−1^.

The seed material was obtained from 20 field populations of *Centaurea cyanus* collected on farms reporting difficulties in cornflower control (Protocol S2). For each population, at least 100 mature infructescences were combined into one sample and air‐dried. After cleaning, seeds were stratified at 4°C for 7 days to break dormancy. The resistance status of each population was preliminarily assessed using a recommended field dose of tribenuron‐methyl (15 g ha^−1^), and weed control was scored visually (0%–100%). Based on these results, six populations were selected for whole‐plant dose–response bioassays.

#### 2.1.1 Whole‐Plant Dose–Response Bioassays

Three susceptible (visual efficacy about 100%) and three potentially resistant to ALS inhibitors (efficacy ≤ 50%) cornflower populations were used as the target plants. Therefore, herbicides [Lumer 50 WG (500 g kg^−1^ of tribenuron‐methyl, ADAMA, Poland) and Saracen 050 SC (50 g L^−1^ of florasulam, Cheminova, Poland)] were used in seven doses. The potentially resistant plants were sprayed with the following doses: N1/2, N1, N2, N4, N8, N16 and N32, where N refers to the field dose (N of tribenuron‐methyl is 15 g ha^−1^ and N of florasulam is 5 g ha^−1^). At the same time, susceptible populations were sprayed with the following herbicide doses: N1/16, N1/8, N1/4, N1/2, N1, N2 and N4. The control plants were the untreated plants (N0). Three weeks after application, the plants were cut at the surface of the soil and fresh weight of the shoots was determined using a Sartorius BP 2000S balance with 0.001 g precision (Sartorius, Göttingen, Germany). ED50 dose, defined as the estimated dose providing 50% control (European Herbicide Resistance Action Committee 2017) was calculated using the ‘drc’ package (Knezevic et al. 2007) in R ver. 4.0.1 (R Core Team 2015). Dose–response curves were plotted using a logarithmic scale for herbicide dose (*X*‐axis).

#### 2.1.2 The Resistance Index (RI)

The RI was used to determine levels of weed resistance to ALS inhibitors.

The RI values were calculated by dividing the ED50 of the potentially resistant biotype with the ED50 of the average of three susceptible biotypes (European Herbicide Resistance Action Committee 2017). The RI classification was based on the modified scale of Beckie and Tardif (2012), which was described by Stankiewicz‐Kosyl et al. (2023).

### 2.2 Preparation of the Plant Material for High‐Throughput Sequencing

For the molecular biology analyses, the second greenhouse experiment was done using plants belonging to biotypes subjected to the whole‐plant dose–response bioassays: three susceptible (ID: S1, S2 and S3) and three resistant (ID: R1, R2 and R3) to ALS inhibitors. Three plants from each biotype were grown in 0.5 L plastic pots in a greenhouse under conditions as specified in section 2.1. At the 2‐leaf stage (BBCH 12), a leaf was taken from each plant, frozen in liquid nitrogen and stored at −80°C.

### 2.3 RNA Extraction and Sequencing

Total RNA extraction, DNA digestion, as well as RNA concentration and purity estimation were performed according to Wrzesińska‐Krupa et al. (2023). RNA quality control was done using the 2100 Bioanalyzer Instrument (Agilent Technologies, Santa Clara, CA, USA). Three samples from susceptible populations and three samples from resistant populations were subjected to sequencing performed by CeGaT GmbH (Tübingen, Germany). One hundred nanogram of total RNA was subjected to library preparation using TruSeq Stranded Total RNA with Ribo‐Zero Plant (Illumina, San Diego, CA, USA), followed by sequencing on the NovaSeq 6000 instrument (Illumina). The sequencing data were deposited into the National Centre for Biotechnology Information (NCBI) Sequence Read Archive (SRA) with the dataset identifiers SRR27672804‐SRR27672795.

### 2.4 Bioinformatic Analysis

Eighteen samples belonging to six biotypes exhibiting different levels of susceptibility to tribenuron methyl, three susceptible (S1–S3) and three resistant (R1–R3), with three biological replicates each, were analysed. Demultiplexing of the sequencing reads was performed with Illumina bcl2fastq (2.20). Adapters were trimmed with Skewer (version 0.2.2) (Jiang et al. 2014). The quality of the FASTQ files was analysed with FastQC (version 0.11.5) (Andrews 2010). The sequencing data were deposited into the National Centre for Biotechnology Information (NCBI) Sequence Read Archive (SRA) with the dataset identifiers SRR27672804‐SRR27672795.

The analysis was performed using two methods. In the first method, the adapter‐trimmed raw forward reads were aligned to the NCBI nr protein database (consisting of sequence data from all organisms (including plants and animals) deposited at the NCBI) (26.11.2019) using Diamond in BLASTX mode (Buchfink et al. 2015). Taxonomic placement was performed using the lowest common ancestor (LCA) algorithm implemented in MEGAN6 Ultimate Edition (version 6.15.2). The percentage of reads classified to the taxonomy analysis with MEGAN6 software differed between samples (44.52%–55.60%). Out of those assigned reads, the reads assigned to the microbial sequences (bacterial, viral, fungal, and archeal) were extracted. Out of them, 23.94%–56.28% were assigned to bacterial taxa and 20.81%–54.61% to viral taxa (Table S1). The relative abundance of taxa within their respective superkingdom was calculated by dividing the number of counts assigned to individual taxa by the number of counts assigned to the relative superkingdom level.

A second method was performed using OmicsBox software (version 3.1.11): Adapter‐trimmed raw forward reads were analysed using the Kraken2 programme (version 2.1.3) (Wood et al. 2019). This programme examines the *k*‐mers within a read and queries a database with those *k*‐mers. The *k*‐mers found in the examined reads were linked to the LCA in the taxonomic tree of all genomes containing that *k*‐mer, using Kraken's genomic library. The database ‘2023_08’ created by OmicsBox consisted of taxa information mainly from Bacteria, Viruses, Archaea and Eukaryota (mostly *Ascomycota*, *Basidiomycota* and *Apicomplexa*). The collection of LCA taxa associated with the *k*‐mers in a read was subsequently examined to generate operational taxonomic units (OTUs). The percentage of reads that were subjected to the evaluation of taxonomic ranks ranged between 24.18% and 44.14%, where 77.10%–89.79% were assigned to bacterial taxa and 0.01%–0.03% to viral taxa (Table S2). The results were sorted by setting the confidence filter to 0.05, similarly to Wielkopolan et al. (2021). The relative abundance of taxa within their respective superkingdom was calculated by dividing the number of reads assigned to individual taxa by the number of reads assigned to the relative superkingdom level. The bacterial and viral taxa were subjected to Differential Abundance Analysis of Taxa between samples derived from resistant and susceptible plants, based on the edgeR package (Robinson et al. 2010). The analysis was performed with the following parameters: Minimum Sample Filter 5, Counts per Million 1, Normalisation Method TMMwsp, Statistical test GLM Likelihood Ratio Test. OTUs displaying *p* < 0.05 and −2 ≥ fold change ≥ 2 in abundance were retained and a clustered heatmap showing differences in their abundances was generated (Škuta et al. 2014). The hierarchical clustering was performed using Euclidean distance calculated between OTUs and the analysed samples.

### 2.5 Phylogenetic Analysis

The nucleotide sequences of the contigs that were the most similar to viruses assigned to the *Partitiviridae* family: melon partitivirus, pepper cryptic virus 1 (PCV1, species: *Deltapartitivirus unocapsici*) and pittosporum cryptic virus 1 (PiCV1) were translated to amino acid (aa) sequences. The resulting sequences (312 aa‐ and 179 aa‐long) were blasted against sequences of other *Partitiviridae* family members taken from the International Committee on Taxonomy of Viruses (ICTV) and also those that were the most similar during analysis in MEGAN6. The overlapping sequence fragments over a length of 179 aa were trimmed and subjected to phylogenetic analysis in MEGA 12 software (Kumar et al. 2024). The analysis included sequence alignment by CLUSTALW (Thompson et al. 1994) followed by phylogenetic tree generation with the Neighbour‐Joining method and Poisson model with 1000 bootstrap replicates.

## 3 Results

### 3.1 Greenhouse Experiments

The conducted study confirmed the susceptibility of the S1–S3 populations to ALS inhibitors. The ED50 values of S biotypes for tribenuron‐methyl were 2.06, 4.73 and 3.43 g ha^−1^ and for florasulam were 1.7, 2.5 and 1.3 g ha^−1^, respectively. The tests also showed a very high resistance of cornflower populations R1, R2 and R3 to tribenuron‐methyl (RRRR—very high resistance, RI > 68.2). This herbicide, used at a dose of up to 32 times higher than the recommended dose, did not cause any damage to the tested populations (ED50 > 480 g ha^−1^). More diverse results were obtained with florasulam. The biotypes showed ED50 values of 2.1, 3.03 and 24.75 g ha^−1^ (Table 1).

**TABLE 1:** Characteristics of cornflower biotypes used in the study. Values reflect the ED50 dose, i.e., the dose causing a 50% shoot fresh weight reduction (g ha^−1^).

Biotype ID	Voivod‐ship	Crop	Type of resistance	HRAC 2—Inhibition of acetolactate synthase					
				Tribenuron‐methyl			Florasulam		
				ED50	RI	Cl	ED50	RI	Cl
S1	Lu	WT	Susceptible	2.06	—	S	1.7	—	S
S2	WM	WW	Susceptible	4.73	—	S	2.5	—	S
S3	Su	WW	Susceptible	3.43	—	S	1.3	—	S
R1	WM	WW	Resistance	> 480	> 140.8	RRRR	2.1	1.15	S
R2	Su	WW	Resistance	> 480	> 140.8	RRRR	3.03	1.66	S
R3	Św	O	Cross‐resistance	> 480	> 140.8	RRRR	24.75	13.11	RRR

*Note:* Field doses of herbicides: tribenuron‐methyl, *N* = 15 g ha^−1^; Florasulam, *N* = 5 g ha^−1^.

Abbreviations: Cl, classification; ED50, the dose causing a 50% reduction of plants' fresh weight (g ha^−1^); RI, the resistance index; Lu, Lubusz; O, oats; Su, Subcarpathian; Św, Świętokrzyskie; WM, Warmian–Masurian; WW, winter wheat; WT, winter triticale.

The performed biotests showed that two populations (R1 and R2) were resistant to only one active ingredient, while one population (R3) showed cross‐resistance (Table 1), i.e., resistance to at least two active ingredients of herbicides belonging to different chemical groups but having the same mechanism of action (Tranel and Wright 2002).

### 3.2 Identification of Viral RNA Sequences in the Transcriptomes of Cornflower Plants Resistant and Sensitive to ALS Inhibitors

#### 3.2.1 RNA Sequences Belonging to the *Partitiviridae*, *Chrysoviridae* and *Endornaviridae* Families Were Identified Using MEGAN6 Software

**FIGURE 1:**
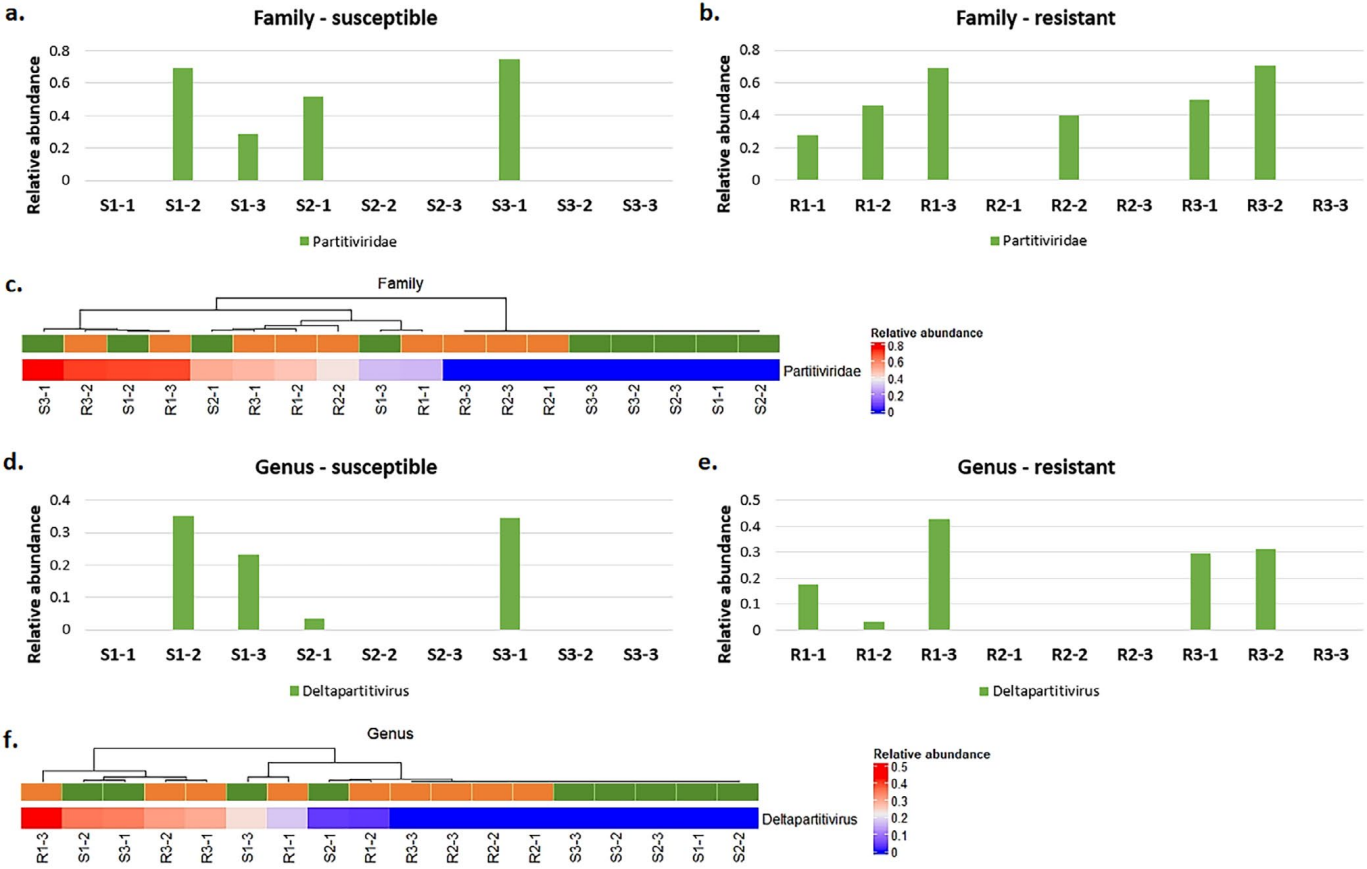
Relative abundance of reads assigned to viral taxa using MEGAN6 software. (a–b, d–e): Virus distribution at the family and genus levels in the samples derived from cornflower plants susceptible and resistant to ALS inhibitors; (c and f) heatmaps based on hierarchical clustering for the relative abundance of viral families and genera identified in samples derived from susceptible (green) and resistant (orange) biotypes. The relative abundance was calculated by dividing the number of counts assigned to individual taxa by the number of counts assigned to the relative superkingdom level.

MEGAN6 software enabled the identification of viral sequences belonging to the *Partitiviridae* (dsRNA viruses), *Chrysoviridae* (dsRNA viruses) and *Endornaviridae* ((+)ssRNA viruses) at the family level, and *Deltapartitivirus*, *Alphachrysovirus* and *Alphaendornavirus* at the genus level, respectively. However, the mean value of relative abundance (calculated by dividing the number of counts assigned to individual taxa by the number of counts assigned to the relative superkingdom level) that were higher than 0.01 concerned only *Partitiviridae* and *Deltapartitivirus* taxa. The counts were found in samples derived from both susceptible (four out of nine samples) and resistant (six out of nine samples) to ALS inhibitors plants (Figure 1a,b,d,e). The relative abundance of those taxa differed between samples within each biotype as well as between biotypes; however, clustering based on a heatmap indicating the relative abundance of virus taxa did not depend on the resistance status of the analysed plants (Figure 1c,f).

#### 3.2.2 RNA Sequences Belonging to the *Solemoviridae*, *Mimiviridae* and *Partitiviridae* Families Were Identified Using Kraken2

**FIGURE 2:**
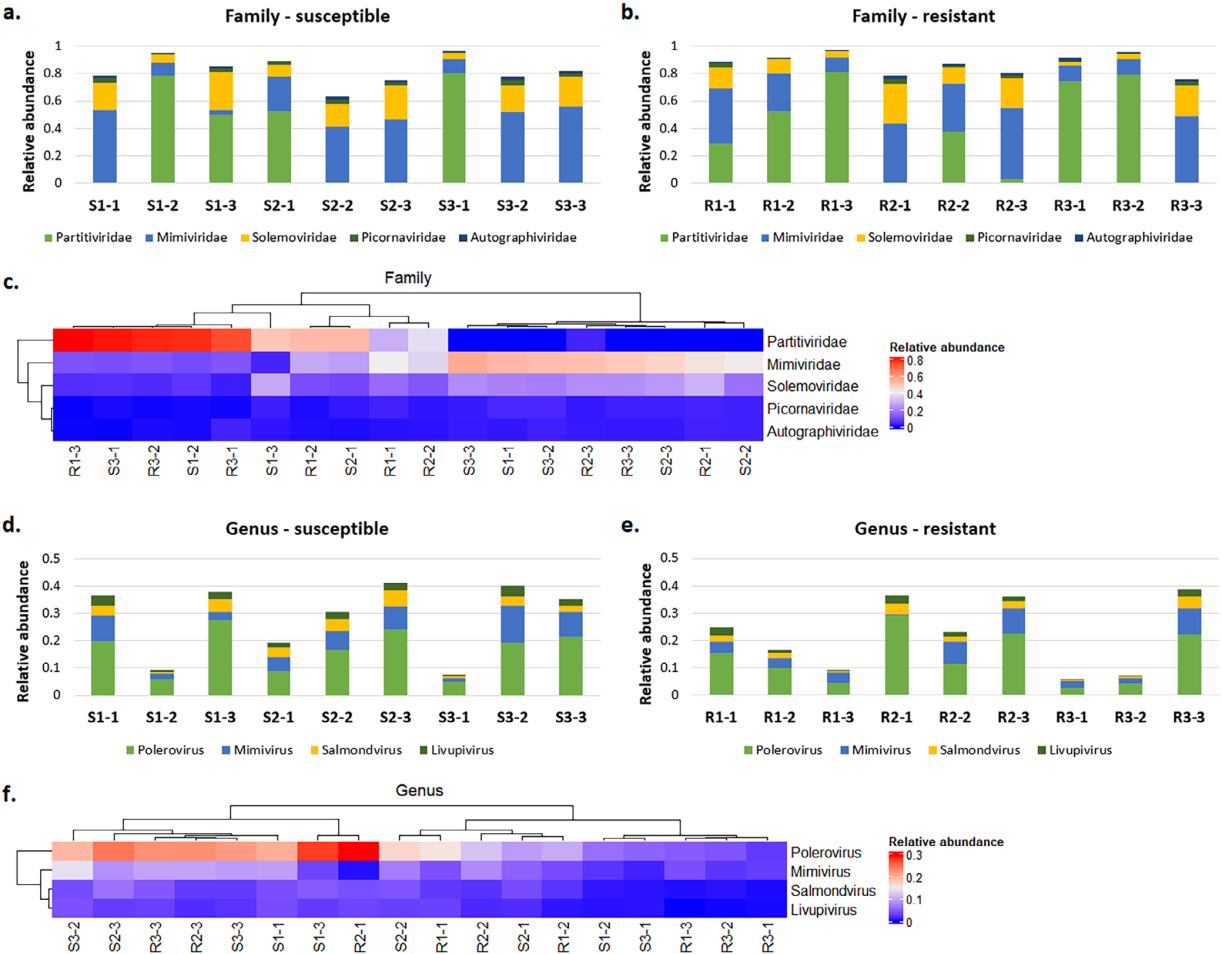
Relative abundance of reads assigned to abundant viral taxa obtained in Kraken2. (a–b, d–e) Virus distribution at the family (top 5) and genus (top 4) levels in the samples derived from cornflower plants susceptible and resistant to ALS inhibitors; (c and f) Heatmaps based on hierarchical clustering for the relative abundance of viral top 5 families and top 4 genera found in two microbial communities derived from susceptible (green) and resistant (orange) plants. The relative abundance was calculated by dividing the number of reads assigned to individual taxa by the number of reads assigned to the relative superkingdom level.

The identification of the viral taxa using Kraken2 revealed the top 5 most abundant families with the highest (> 0.01) mean value of relative abundances of reads assigned to individual OTUs calculated for all samples, including *Solemoviridae* ((+)ssRNA genome), *Mimiviridae* (dsDNA) and *Partitiviridae* being the most abundant across viral taxa on the family level (Figure 2a–c). *Livupivirus* ((+)ssRNA virus in the family *Picornaviridae*), *Salmondvirus*, *Mimivirus* and *Polerovirus* ((+)ssRNA virus in the family *Solemoviridae*) were pinpointed as the top 4 most abundant viral genera (mean value of relative abundance > 0.01), with the latter one as the most abundant (Figure 2d–f). The relative abundances of those genera differed between samples within biotypes and between biotypes susceptible and resistant to tribenuron‐methyl; however, those differences were not dependent on the resistance status on both family and genus levels (Figure 2c,d).

#### 3.2.3 The Identification of Sequence Fragments of Two Previously Undescribed Viruses Belonging to the Family *Partitiviridae*

**FIGURE 3:**
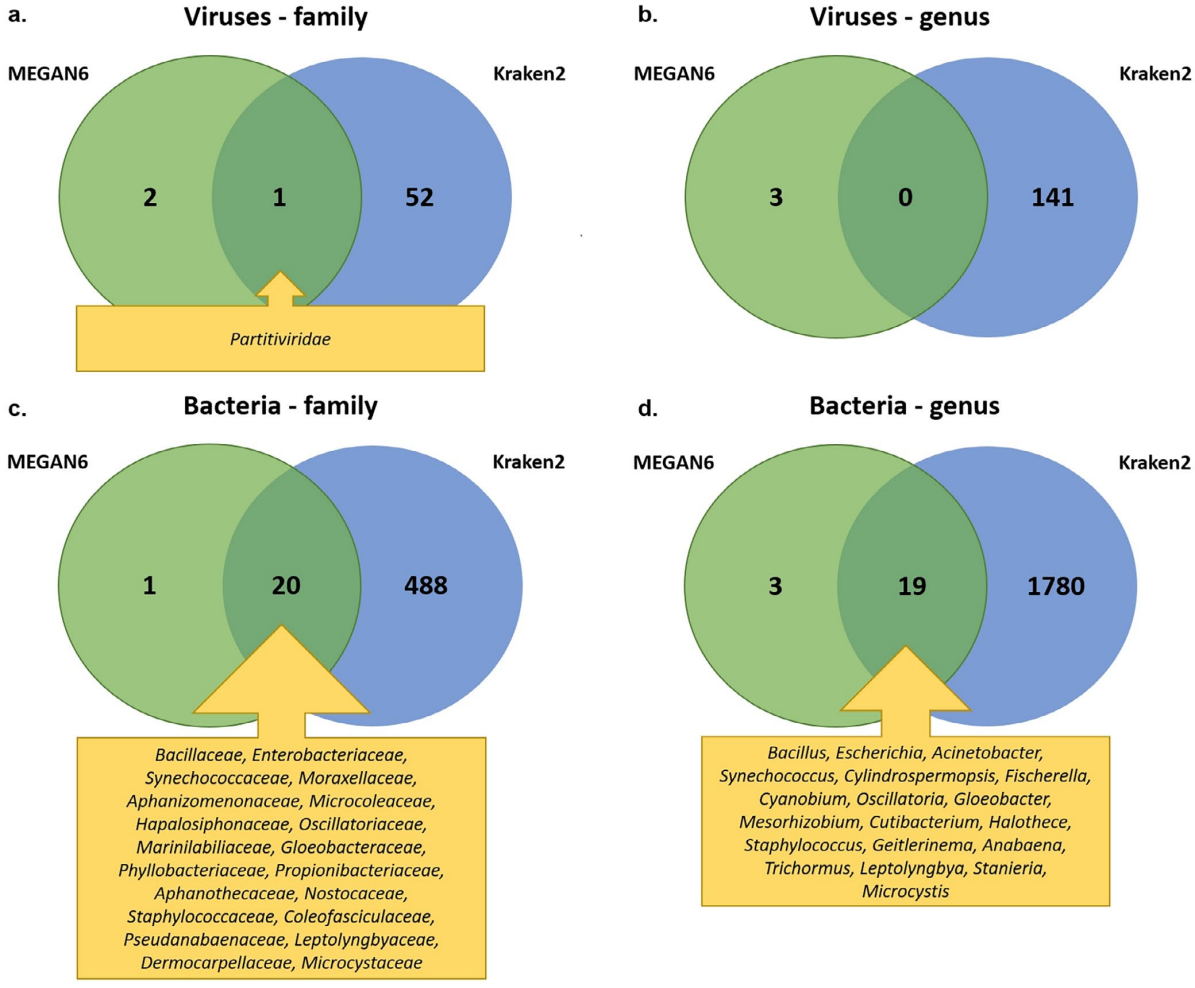
Comparison of the identified viral (a and b) and bacterial (c and d) taxa at family and genus levels by MEGAN6 and Kraken2 tools.

**FIGURE 4:**
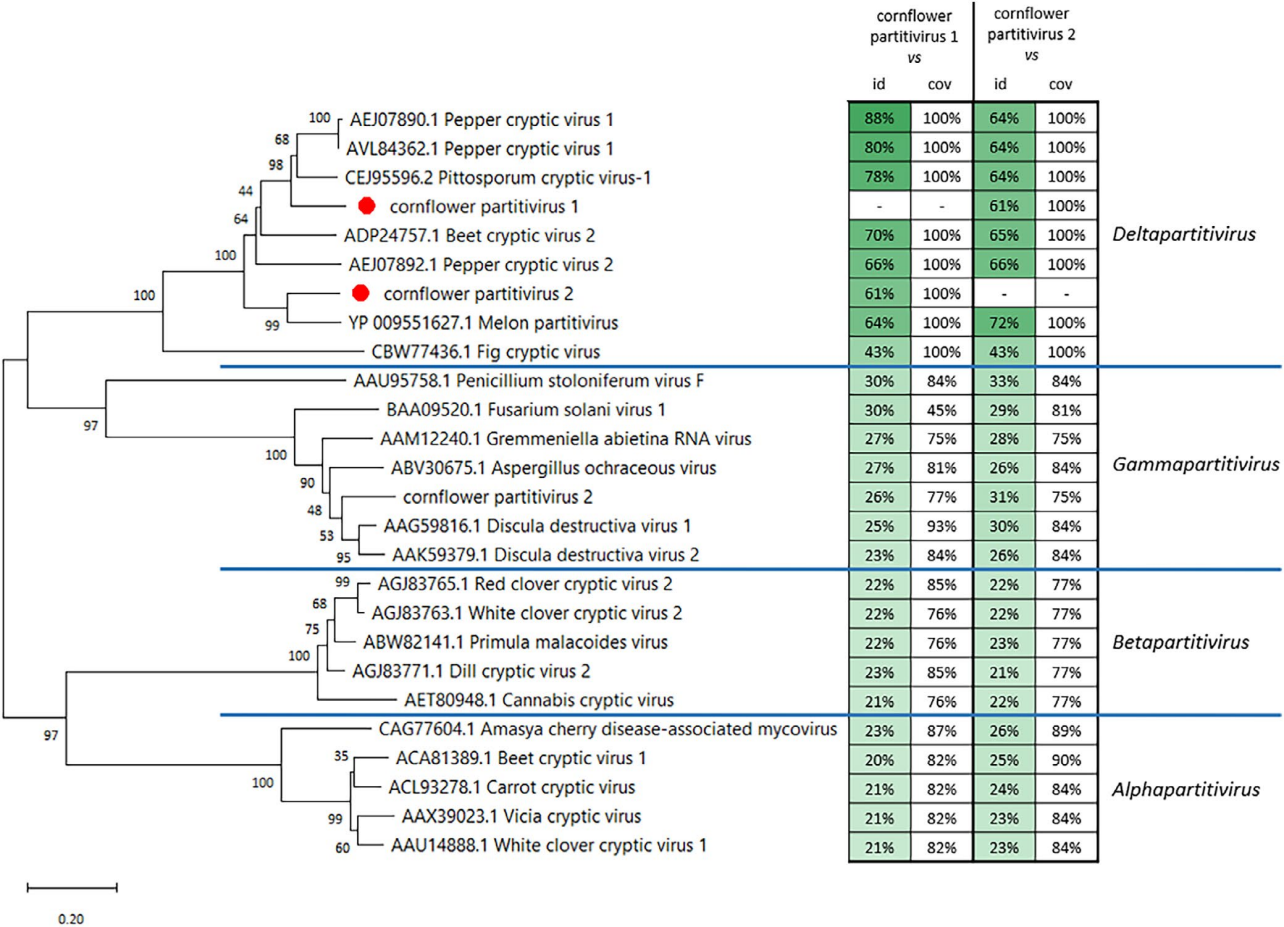
Phylogenetic analysis of 179 amino acid sequence fragments of RNA‐dependent RNA polymerase from viruses belonging to the *Partitiviridae* family. Here, tentatively named cornflower partitivirus 1 and cornflower partitivirus 2 were marked with red dots. The representative viral sequences from classified members of the family *Partitiviridae* were taken from the International Committee on Taxonomy of Viruses (ICTV), as well as the most similar to cornflower partitivirus 1 and cornflower partitivirus 2 sequences found in MEGAN6 analysis. The table presents amino acid sequence fragments comparison (id—sequence identity, cov—sequence coverage) between cornflower partitivirus 1 or cornflower partitivirus 2 and viral sequences taken to phylogenetic analysis performed with NCBI BLAST. The scale bar represents genetic distance. The green colouring of the ‘id’ columns corresponds with the percentage value of sequence identity. The higher value the more intense colour.

Both methods led to the detection of sequences of viruses belonging to the *Partitiviridae* family; however, the lower taxon level—*Deltapartitivirus* was absent at the genus level in the Kraken 2 results (Figure 3a,b), which may imply the presence of an unclassified *Partitiviridae* at the genus level or the presence of previously undescribed virus species. Based on the MEGAN6 results, the identified contigs belong to the *Deltapartitivirus* genus, and at the species level, those contigs showed the highest sequence similarity to melon partitivirus, PCV1 and PiCV1. A more detailed analysis showed that the sequences of contigs similar to sequences of PCV1 and PiCV1 were the same. Finally, there are groups of contigs constituting the fragments of sequences coding for RNA‐dependent RNA polymerases of two viruses belonging to the family *Partitiviridae,* tentatively named here as cornflower partitivirus 1 (941‐nt sequence fragment deposited at GenBank with accession number PV571748) and cornflower partitivirus 2 (537‐nt sequence fragment deposited at GenBank with accession number PV571749). These nucleotide sequences were translated to amino acid sequences, resulting in 313 aa‐ and 179 aa‐long fragments for cornflower partitivirus 1 and cornflower partitivirus 2, respectively. To determine to which genus within the *Partitiviridae* family those viruses belong, a phylogenetic tree was generated (Figure 4). This analysis confirmed that both viruses belong to the *Deltapartitivirus* genus. Cornflower partitivirus 1 clusters with PCV1 and PiCV1 and has 43%–88% sequence identity with viruses belonging to the *Deltapartitivirus* genus, while cornflower partitivirus 2 clusters with melon partitivirus and has 43%–72% sequence identity with viruses belonging to the *Deltapartitivirus* genus.

In the next stage of the analysis, it was verified whether the resistance status of cornflower plants affects the presence of viruses. It was found that the presence of the viral taxa and their relative abundance are not correlated with the resistance status to ALS inhibitors of cornflower plants based on the identified reads corresponding to viral genomic sequences in the analysed samples. The performed Differential Abundance Analysis of Taxa displayed no statistically significant results (*p* < 0.05) with −2 ≥ fold change ≥ 2.

### 3.3 Transcriptomic Data Shows a Differential Occurrence of Bacteria in Populations Sensitive and Resistant to ALS Inhibitors

**FIGURE 5:**
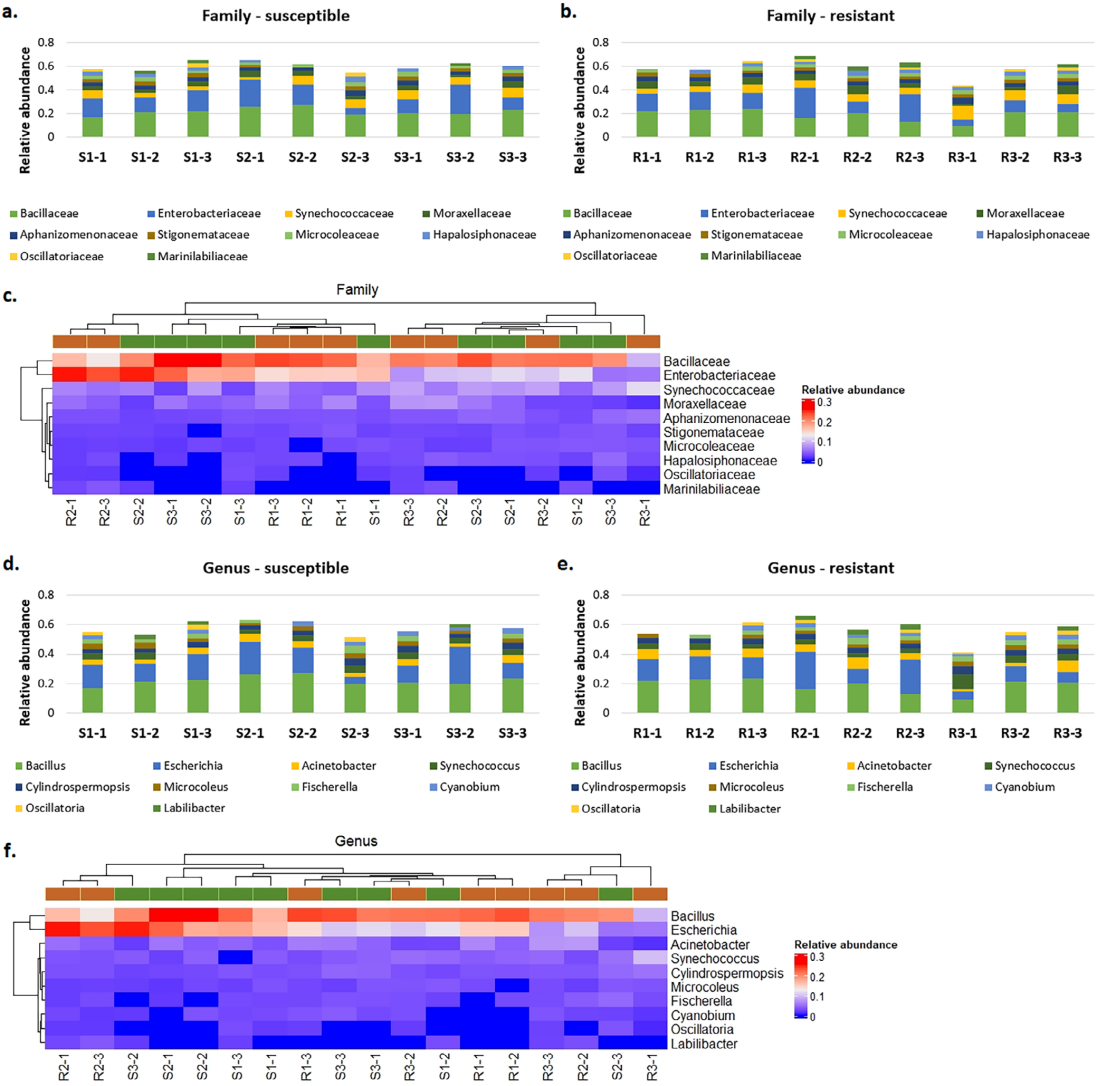
Relative abundance of reads assigned to bacterial taxa using MEGAN6 software. (a–b, d–e) Bacteria distribution at the family and genus levels in the samples derived cornflower plants susceptible and resistant to ALS inhibitors; (c and f) Heatmaps based on hierarchical clustering for the relative abundance of bacterial families and genera identified in samples derived from susceptible (green) and resistant (orange) biotypes. The relative abundance was calculated by dividing the number of counts assigned to individual taxa by the number of counts assigned to the relative superkingdom level.

Among the bacterial families detected at the highest relative abundance level using MEGAN6 were *Bacillaceae* and *Enterobacteriaceae* among 10 bacterial families displaying mean relative abundance higher than 0.01 (Figure 5a–c), while at the genus level, *Bacillus* and *Escherichia* were out of 10 bacterial taxa (Figure 5d–f). The relative abundance of counts ascribed to those taxa cannot differentiate between samples derived from plants susceptible and resistant to ALS inhibitors (Figure 5c,d).

**FIGURE 6:**
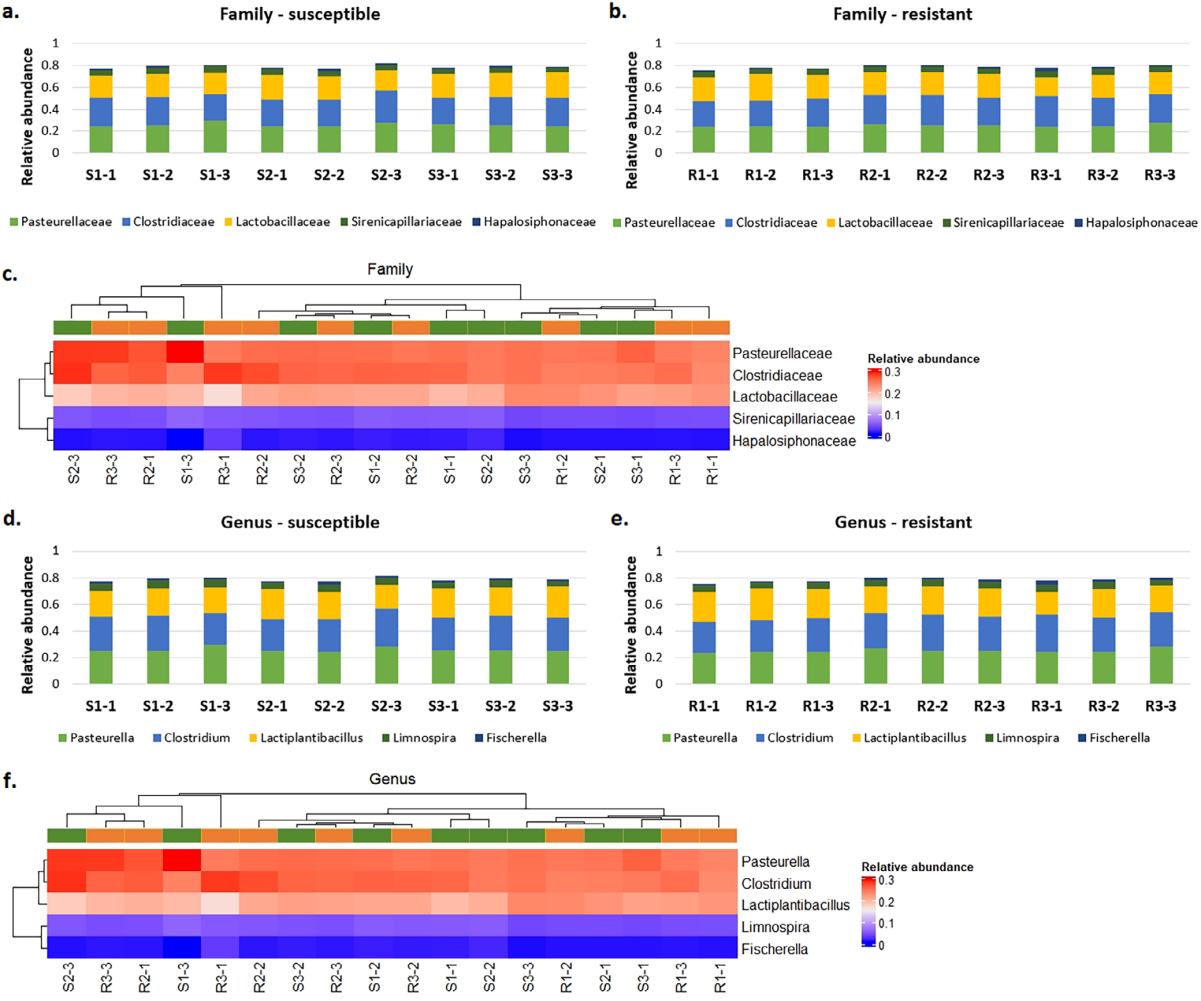
Relative abundance of reads assigned to the top 5 most abundant bacterial taxa obtained in Kraken2. (a–b, d–e) Bacteria distribution at the family and genus levels in the samples derived from cornflower plants susceptible and resistant to ALS inhibitors; (c and f) Heatmaps based on hierarchical clustering for the relative abundance of bacterial families and genera found in two microbial sequences present in susceptible (green) and resistant (orange) plants. The relative abundance was calculated by dividing the number of reads assigned to individual taxa by the number of reads assigned to the relative superkingdom level.

The most abundant bacterial families (mean relative abundances of reads assigned to individual OTUs > 0.01) identified using Kraken2 were those belonging to *Pasteurellaceae*, *Clostridiaceae* and *Lactobacillaceae* (Figure 6a–c). Whereas the most abundant bacterial genera (> 0.01 mean value of relative abundances of reads assigned to individual OTU) were *Pasteurella*, *Clostridium* and *Lactiplantibacillus* (Figure 6d–f). However, there are no significant differences in the relative abundances of those reads between samples and between biotypes.

The comparison of the identified bacterial taxa using MEGAN6 and Kraken2 analyses resulted in the number of shared families (20) and genera (19); however, the highly abundant bacterial taxa (both families and genera) found using MEGAN6 were not present among the most abundant (> 0.01 mean value of relative abundances of OTUs) bacterial taxa obtained using Kraken2 (Figure 3c,d).

**FIGURE 7:**
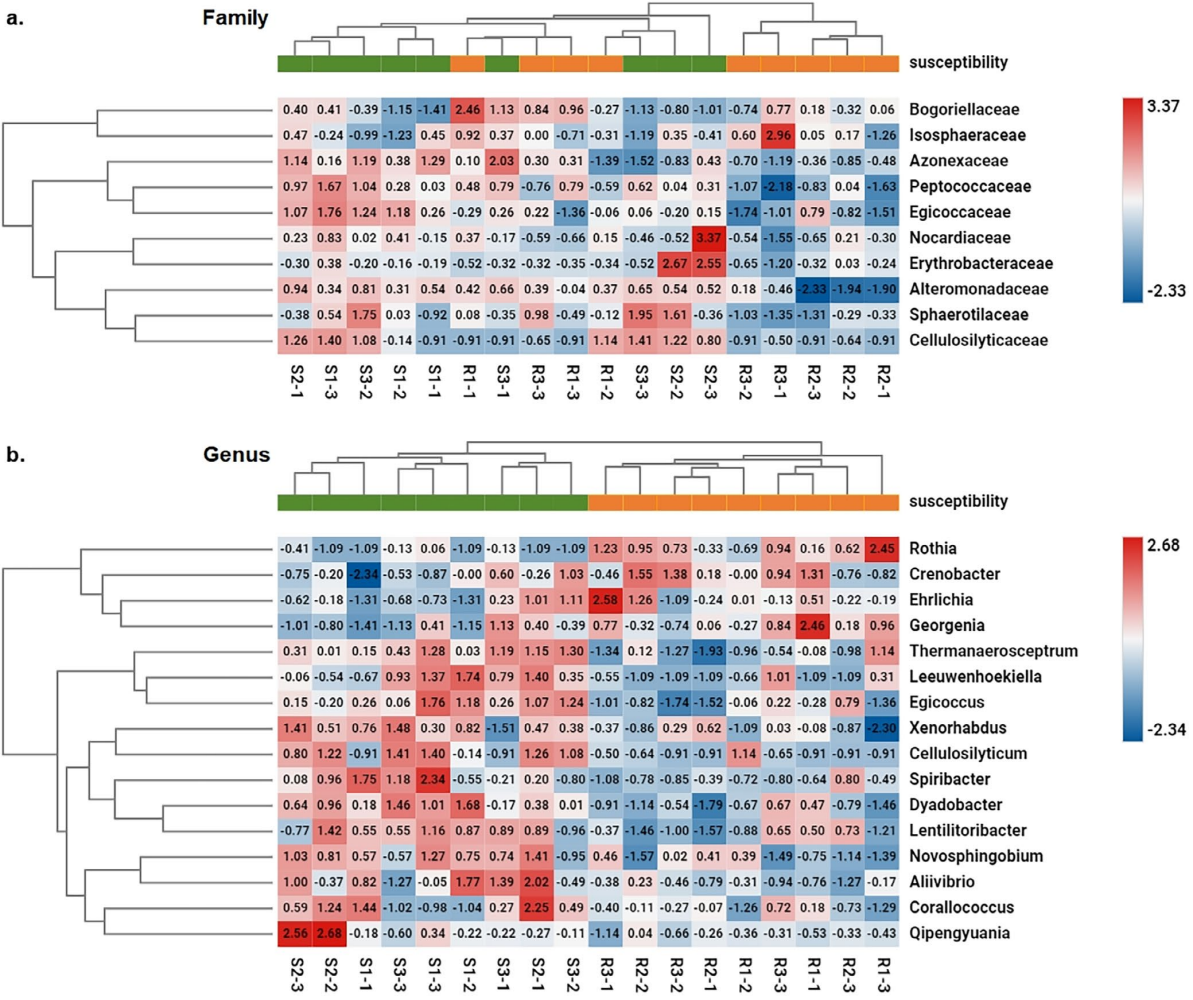
Heatmap of significantly differing abundancies of bacterial OTUs classified at family (a) and genus (b) level between two microbial communities derived from susceptible (green) and resistant (orange) plants (*p* < 0.05, −2 ≥ fold change ≥ 2). Differential Abundance Analysis of Taxa performed between the abundances of reads assigned to individual taxa identified in resistant versus sensitive to ALS inhibitors cornflower plants. The hierarchical clustering was performed using Euclidean distance calculated between treads assigned to individual taxa and the analysed samples. The scale bar represents the abundance values. The fold change values obtained in Differential Abundance Analysis of bacterial reads classified at family and genus levels between resistant and susceptible plants are presented in Table S3.

Differential Abundance Analysis of Taxa revealed 10 bacterial families displaying statistically different abundances in reads assigned to individual OTUs between samples derived from plants susceptible and resistant to ALS inhibitors; however, the samples did not cluster according to the resistance status (Figure 7a). The same analysis performed at the bacterial genus level showed 16 taxa with statistically different abundances in reads assigned to individual OTUs between resistant and susceptible tribenuron‐methyl cornflower plants (Figure 7b). Those genera were not among the most abundant taxa obtained with Kraken2, but they clustered according to the resistance status.

## 4 Discussion

Herbicides that target ALS, a plant enzyme involved in the biosynthesis of branched‐chain amino acids and essential for protein synthesis, are among the most widely used in the world because they are relatively inexpensive, effective at low doses, and have low toxicity to mammals (Dayan et al. 2019; Tranel and Wright 2002).

Building on this, one of the weed species known to exhibit resistance to ALS inhibitors is cornflower. Stankiewicz‐Kosyl et al. (2021) studied 159 populations of potentially herbicide‐resistant cornflower in Poland in 2017–2020. Biological experiments confirmed resistance in 28 biotypes to tribenuron‐methyl and 8 to florasulam. Preliminary tests verifying resistance to tribenuron methyl in this study enabled the selection of three populations with very high resistance (RRRR) to this active ingredient. In the experiment on the calculation of ED50 for tribenuron‐methyl and florasulam, cross‐resistance to both herbicides was confirmed in one population.

A recent report indicates that several weed species of *Centaurea* ( *C. cyanus* L., *C. diluta*, *C. melitensis* and *C. pullata* ) exhibit tolerance to multiple ALS‐inhibiting herbicides, including four sulfonylurea compounds (Palma‐Bautista et al. 2023), which is consistent with our findings of ALS‐resistant *C. cyanus* populations in Poland and further highlights the risk of selecting highly resistant biotypes under intensive use of herbicides from this group. The populations for those studies were collected in cereals of the southern half of the Iberian Peninsula, where these herbicides have never been applied. *C. diluta* was the most tolerant species to the tribenuron‐methyl treatment (ED50 = 146.5 g ai ha^−1^), while the ED50 of *C. cyanus* was 9.6 g ai ha^−1^ (Palma‐Bautista et al. 2023). It was indicated that the tolerance was governed by CYP450, leading to enhanced herbicide detoxification (Palma‐Bautista et al. 2023). This study clearly shows that both types of cornflower populations with susceptibility and resistance to tribenuron‐methyl occur in Polish fields. The ED50 of susceptible populations of this weed species was in the range of 13%–31% of the field dose (15 g ai ha^−1^). However, the RI for the three populations (R1–R3) was above 140.76, which classifies them in the group with a very high level of resistance—RRRR—much higher than that reported in tolerant cornflower populations reported from the Iberian Peninsula.

In addition to the intrinsic plant mechanisms contributing to the development of resistance, increasing attention is being paid to the contribution of the plant‐associated microbial community in coping with abiotic stresses. In the case of pesticide resistance, there are many examples of the host microbiota playing an important role, especially in the case of insect‐associated bacteria involvement in insecticide resistance (Kikuchi et al. 2012; Wielkopolan et al. 2024). Microorganisms on plants can be harmful but also beneficial to plants, and in many cases, can increase resistance to abiotic stresses (Caddell et al. 2019; Govindasamy et al. 2018; Motamedi et al. 2022). This has been demonstrated for the bacterial microbiome and is also postulated for some viruses.

*C. cyanus* has not yet been studied in this respect, so based on obtained transcriptome datasets for cornflower plants belonging to populations susceptible and resistant to ALS inhibitors, we extracted sequences that may belong to bacteria and viruses, followed by an assessment of the prevalence using two algorithms dedicated to these purposes, MEGAN6 and Kraken2. Previous studies showed that there are frequent discrepancies as a result of analyses using each of these algorithms (Xu et al. 2023). Discrepancies in microbial taxonomic assignment and abundance profiles were also observed in the analysis of the metagenome and metatranscriptome of overwintering pepper fruits, where MEGAN6 and Kraken2 were applied (Jo et al. 2021). When Kraken2 was used, the analysis yielded a higher number of identified organisms, which may result from the differences in database content and the read profiling algorithms employed by the two programmes (Xu et al. 2023). Similar discrepancies were observed in our study. Among viral sequences, only one taxon at the family level was identified by both algorithms. In bacterial analyses, we also detected numerous inconsistencies between the two methods, and the largest differences occurred among the most abundant taxa, although the overall OTU coverage for bacterial sequences remained considerable.

Importantly, in datasets of this study, we observed viral sequences belonging to non‐plant hosts, such as those of the bacteriophage *Samondvirus* genus or viruses of insects. The same situation concerned bacterial sequences identified since they can come from soil and water contaminations present in the harvested plants.

Viruses of the phytobiomes are still poorly studied, especially viruses of wild plants as well as of organisms that interact with plants (Roossinck 2019). However, interest in studying weed viruses, especially as the reservoir of crop pathogens and due to their importance for host plant biology, has been growing in recent years. There are viruses whose presence in a plant can be beneficial to its host. Examples of such beneficial phytobiome viruses were described by Roossinck (2019). It was also shown that even acute plant viruses (e.g., tobacco mosaic virus, brome mosaic virus or cucumber mosaic virus) can improve drought or heat resistance (Anfoka et al. 2016; Corrales‐Gutierrez et al. 2020; Shteinberg et al. 2021; Xu et al. 2008). In this context, we asked whether there are viral sequences mostly associated with resistant biotypes of cornflower plants. The analyses of this study revealed the presence of viruses from the family *Partitiviridae* (by both methods) previously identified in the weeds (Rivarez et al. 2023; Sabbadin et al. 2017). Using MEGAN6, we were able to identify them on the genus level as belonging to the *Deltapartitivirus*. Additionally, solemo‐ and poleroviruses were indicated by using Kraken2, and they all were also previously identified in the weeds (Rivarez et al. 2023; Sabbadin et al. 2017; Umar et al. 2022). Interestingly, Sabbadin et al. (2017) pointed out in their research that many populations of black‐grass infected with partitiviruses showed NTS resistance. However, based on the data collected on plants belonging to six cornflower populations, we cannot conclude that there is a correlation between the occurrence of specific viruses and the increased resistance to herbicide stress. Similarly, in the approach used by Sabbadin et al. (2017), where analyses of resistant and susceptible biotypes of black‐grass showed the presence of undescribed viruses belonging to the *Partitiviridae* and *Rhabdoviridae* families, no direct causative link was established linking viral infection to herbicide resistance. We found out that the viruses present in cornflower plants were present in both resistant and susceptible biotypes, and their abundance was sometimes comparable. Thus, more biotypes need to be analysed to clarify this issue. Additionally, downstream studies will be needed to identify viruses at the species level.

A different situation was observed in the case of cornflower‐associated bacterial sequences. Namely, at the genus level, resistant and susceptible populations created two separate clusters on the heatmap obtained based on the differential abundance analysis of reads assigned to individual OTUs depending on the resistance status. Thus, it may be that the cornflower‐associated bacteria contribute to their resistance status. Additionally, based on the clustering of cornflower populations, it can be hypothesised that here not individual bacteria but several bacterial taxons present together and at certain abundance levels may contribute to (or simply correlate with) increased herbicide resistance in this plant species. However, as with viruses, such a conclusion based on the analysed biotypes would be premature. It would be necessary to analyse a larger sample size, in addition to looking at the occurrence of specific taxa and elucidating their possible role in the detoxification of active substances of herbicides.

Nevertheless, based on the differential abundance analysis between susceptible and resistant biotypes, several bacterial genera were found as important: *Rothia*, *Georgenia*, *Ehrlichia*, *Crenobacetr*, *Thermanaerosceptrum*, *Egicoccus*, *Xenorhabdus*, *Corallococcus*, *Dyadobacter*, *Lentilitoribacter*, *Novosphingobium*, *Leeuwenhoekiella*, *Aliivibrio*, *Cellulosilyticum*, *Qipengyuani*, *Spiribacter*, but all were relatively low abundant in general. Some of them were described in plants, also as plant growth‐promoting (e.g., *Novosphingobium* or *Dyadobacter*), while others were found in water and wastewater (Kumar et al. 2018; Vives‐Peris et al. 2018). The highest differences in abundance in favour of resistant plants were detected in the case of *Rothia*, the genus prevalent in all cornflowers of herbicide‐resistant biotypes, and in about half of the cornflowers of susceptible biotypes (12.2‐fold change, Table S3). Endophytic *Rothia* strains of different species have been reported from the rhizosphere and in tissues of several plants, including maize, *Vaccinium myrtillus* , *Santalum album* or Arabidopsis (Mažeikienė et al. 2021; Pisarska and Pietr 2015; Sokolov et al. 2020; Tuikhar et al. 2022). On the contrary, the abundance of sequences belonging to *Aliivibrio*, *Cellulosilyticum*, *Qipengyuani* and *Spiribacter* was significantly lower in plants belonging to herbicide‐resistant than in susceptible biotypes (fold change abundance < −5, Table S3), and present in all or almost all plants. The latter genus was found in the intermediate salinity zones of hypersaline environments (León et al. 2014, 2020).

The most abundant bacterial genera obtained in Kraken2 analysis were *Pasteurella*, *Clostridium* and *Lactiplantibacillus*. *Clostridium* was found to enhance salinity tolerance in *Miscanthus sinensis* (Ye et al. 2005), and together with *Pasteurella* is one of the rhizobacteria functioning as plant growth‐promoting (Ghayoumi et al. 2022; Pant et al. 2016). Moreover, *Pasteurella* was shown to efficiently contribute to rhizoremediation (Pant et al. 2016). *Lactiplantibacillus* is widely present in multiple niches and has antagonistic activity against pathogens infecting different crops (Daranas et al. 2019).

Among the bacterial genera found, 19 were found in cornflower by both MEGAN6 and Kraken2. These are *Bacillus*, *Acinetobacter*, *Synechococcus*, *Escherichia*, *Cylindrospermopsis*, *Fischerella*, *Cyanobium*, *Oscillatoria*, *Gloeobacter*, *Mesorhizobium*, *Cutibacterium*, *Halothece*, *Staphylococcus*, *Geitlerinema*, *Anabaena*, *Trichormus*, *Leptolyngbya*, *Stanieria* and *Microcystis*. Species belonging to some of these genera are known to be beneficial, plant growth‐promoting and exert positive effects on soil and plant health. This especially concerns *Bacillus* and *Mesorhizobium*, but also *Acinetobacter* (Gulati et al. 2009; Laranjo et al. 2014; Saxena et al. 2020). Additionally, species belonging to these genera were found to participate in the detoxification of harmful substances or alleviate external abiotic conditions (Ali et al. 2023; Chi et al. 2022; Irshad et al. 2020; Yamaga et al. 2010). Many of the commonly found (by both methods) genera occurring in *C. cyanus* are cyanobacteria (e.g., *Cyanobium*, *Synechococcus*, *Fischerella*, *Gloeobacter*). Interestingly, some cyanobacteria are able to establish symbiotic relationships with plants in various endophytic and epiphytic associations (Álvarez et al. 2023). However, none of the above‐mentioned genera were found among those observed on the heatmap done based on the differential abundance analysis of reads assigned to individual OTUs between resistant and susceptible plants and clustering separately depending on the cornflower resistance status (Figure 6).

## 5 Conclusions

This study provides the first insight into the viral and bacterial communities associated with *Centaurea cyanus* plants differing in their susceptibility to ALS‐inhibiting herbicides. Using a metatranscriptomic approach, sequences of viral origin—including two previously uncharacterised members of the *Partitiviridae* family—as well as numerous sequences associated with bacterial genera with potential ecological relevance were identified. Although no direct correlation between viral presence and herbicide resistance was detected, clear differences in bacterial composition were observed between susceptible and resistant biotypes. The clustering of resistant populations based on specific bacterial taxa suggests a possible contribution of the microbiome to NTSR mechanisms. Several genera, including *Rothia*, *Novosphingobium* and *Dyadobacter*, are known to enhance plant stress tolerance or facilitate xenobiotic degradation, raising the hypothesis that certain microbial consortia may support herbicide detoxification or plant resilience under chemical stress. As weeds are recognised reservoirs of both phytopathogens and beneficial microorganisms, characterising their microbiome may further help predict plant–microbe–herbicide interactions and the broader ecological consequences of pesticide use.

## Author Contributions

**Katarzyna Marcinkowska:** data curation, formal analysis, investigation, methodology, resources, funding acquisition, project administration, visualisation, writing – original draft, writing – review and editing. **Barbara Wrzesińska‐Krupa:** data curation, formal analysis, investigation, methodology, resources, validation, visualisation, software, writing – original draft, writing – review and editing. **Aleksandra Obrępalska‐Stęplowska:** conceptualization, data curation, investigation, methodology, resources, supervision, writing – original draft, writing – review and editing.

## Funding

This work was supported by National Science Centre, Poland (2020/04/X/NZ9/01767).

## Conflicts of Interest

The authors declare no conflicts of interest.

## Supporting information


**TABLE S1:** Percentages of reads assigned to main microorganism taxa belonging to Bacteria, Archaea, Eukaryota (Fungi) and Viruses as well as not assigned reads obtained in MEGAN6. S—susceptible plants, R—resistant plants, S/R(1–3)—the number of a biotype, S/R(1–3)‐(1–3)—the number of a sample.
**TABLE S2:** Percentages of reads assigned to Bacteria, Archaea, Eukaryota and Viruses as well as not assigned reads obtained in Kraken2. S—susceptible plants, R—resistant plants, S/R(1–3)—number of biotype, S/R(1–3)‐(1–3)—number of sample.
**TABLE S3:** Significantly differing reads abundancies of bacterial OTUs classified at family (a) and genus (b) level between two microbial communities derived from susceptible and resistant plants (*p* < 0.05, −2 ≥ fold change ≥ 2). Differential Abundance Analysis of Taxa performed between the abundances of reads assigned to individual OTUs identified in resistant versus sensitive to ALS inhibitors cornflower plants. Taxa presence in the analysed samples (out of nine samples for each condition) is presented.
**PROTOCOL S1**. Greenhouse experiments.
**PROTOCOL S2**. Test verifying plant populations for studies.

## Data Availability

The data that support the findings of this study are available within the article and its Supporting Information and from the corresponding author upon reasonable request. The sequencing data were deposited into the National Centre for Biotechnology Information (NCBI) Sequence Read Archive (SRA) with the dataset identifiers SRR27672804‐SRR27672795. Sequence fragments of tentatively named as cornflower partitivirus 1 and cornflower partitivirus 2 were deposited at GenBank with accession numbers PV571748 and PV571749, respectively.
