# Supplementary material for: Insights Into Sequences of Viral and Bacterial Origin in the Metatranscriptome of Centaurea cyanus L. Susceptible and Resistant to Acetolactate Synthase (ALS)‐Inhibiting Herbicides
Source: Environ Microbiol Rep. 2026 Feb 5;18(1):e70287. doi: 10.1111/1758-2229.70287 (PMC12874495; doi:10.1111/1758-2229.70287)
Supplement: Supplementary file 1 — TABLE S1: Percentages of reads assigned to main microorganism taxa belonging to Bacteria, Archaea, Eukaryota (Fungi) and Viruses as well as not assigned reads obtained in MEGAN6. S—susceptible plants, R—resistant plants, S/R(1–3)—the number of a biotype, S/R(1–3)‐(1–3)—the number of a sample. TABLE S2: Percentages of reads assigned to Bacteria, Archaea, Eukaryota and Viruses as well as not assigned reads obtained in Kraken2. S—susceptible plants, R—resistant plants, S/R(1–3)—number of biotype, S/R(1–3)‐(1–3)—number of sample. TABLE S3: Significantly differing reads abundancies of bacterial OTUs classified at family (a) and genus (b) level between two microbial communities derived from susceptible and resistant plants (p < 0.05, −2 ≥ fold change ≥ 2). Differential Abundance Analysis of Taxa performed between the abundances of reads assigned to individual OTUs identified in resistant versus sensitive to ALS inhibitors cornflower plants. Taxa presence in the analysed samples (out of nine samples for each condition) is presented. PROTOCOL S1. Greenhouse experiments. PROTOCOL S2. Test verifying plant populations for studies. [file EMI4-18-e70287-s001.docx]

**Supporting Information**

**Insights into sequences of viral and bacterial origin in the metatranscriptome of *Centaurea cyanus* L. susceptible and resistant to acetolactate synthase (ALS)-inhibiting herbicides**

Katarzyna Marcinkowska^1,*^ (https://orcid.org/0000-0002-3909-6383), Barbara Wrzesińska-Krupa^2,*^ (https://orcid.org/0000-0002-2142-9362), Aleksandra Obrępalska-Stęplowska^2^ (https://orcid.org/0000-0002-0314-8110)

1. Institute of Plant Protection – National Research Institute, Department of Weed Science and Plant Protection Techniques, Węgorka 20, 60-318 Poznań, Poland
2. Institute of Plant Protection – National Research Institute, Department of Molecular Biology and Biotechnology, Węgorka 20, 60-318 Poznań, Poland

^*^ These authors contributed equally to this work.

**Correspondence:** Katarzyna Marcinkowska ([k.marcinkowska@iorpib.poznan.pl](mailto:k.marcinkowska@iorpib.poznan.pl)), Aleksandra Obrępalska-Stęplowska ([ao.steplowska@iorpib.poznan.pl](mailto:ao.steplowska@iorpib.poznan.pl))

**Protocol S1:** Greenhouse experiments

All biological studies were performed in a greenhouse with controlled environmental conditions: a temperature of 20 (±2) °C, humidity of 60% and photoperiod of 16/8 day/night hours. All plants were grown in 0.5 L plastic pots containing commercial peat-based potting material (Kronen, Lasland sp. z o.o. Grądy, Poland). The seedlings were thinned to five per pot within 10 days after emergence and watered as needed for healthy growth (Marcinkowska *et al.* 2023, Pernak *et al.* 2022).

The applications were made using a moving nozzle sprayer delivering 200 L ha^-1^ of spray solution from a flat-fan TeeJet 1102 nozzle (TeeJet Technologies, Wheaton, IL, USA) at 0.2 MPa operating pressure. The nozzle was positioned at 40 cm from the tops of plants and moved at a constant speed of 3.1 m s^-1^. The plants were treated at the 2-leaf stage, BBCH 12 with a water solution of herbicides. Directly after application, the plants were placed in the greenhouse under the conditions described above. All experiments were performed as a completely randomized setup with three replications per treatment.

**Protocol S2:** Test verifying plant populations for studies

The twenty seed samples of cornflower (*Centaurea cyanus* L.) were collected on farms where farmers reported cornflower control difficulties. At least 100 fully developed infruitescences of cornflower were harvested from a few spots (a plot of 100 × 50 m) and combined into 1 sample in a paper bag (Burgos *et al.* 2013, European Herbicide Resistance Action Committee 2017, Panozzo *et al.* 2015, Stankiewicz-Kosyl *et al.* 2021). The collected samples were air-dried in a ventilated, dry room and the samples were then cleaned. To break dormancy, the seeds were kept in a fridge at 4 °C for 7 days (Panozzo *et al.* 2015). Then, the resistance trait was confirmed in the given populations. In these studies, cornflower plants were sprayed with the recommended dose of tribenuron-methyl (N=15 g ha^-1^). Weed control was evaluated visually using a scale of 0 (no effect) to 100% (complete weed destruction) three weeks after application. Six populations collected from various locations in Poland were qualified for whole-plant dose-response bioassays.

**Table S1.** Percentages of reads assigned to main microorganism taxa belonging to Bacteria, Archaea, Eukaryota (Fungi) and Viruses as well as not assigned reads obtained in MEGAN6. S – susceptible plants, R – resistant plants, S/R(**1-3**) – the number of a biotype, S/R(1-3)-(**1-3**) – the number of a sample

| Sample | Number of raw reads (in million) | Assigned reads to all taxonomic ranks [%] | Reads assigned to microorganismal taxa [%] | | | |
| --- | --- | --- | --- | --- | --- | --- |
|  |  |  | **Bacteria** | **Archaea** | **Eukaryota** | **Viruses** |
| S1-1 | 69.140 | 49.39 | 45.19 | 0 | 29.69 | 25.13 |
| S1-2 | 45.531 | 50.14 | 28.70 | 0 | 22.88 | 48.42 |
| S1-3 | 84.762 | 55.60 | 56.28 | 0 | 4.91 | 38.81 |
| S2-1 | 47.771 | 49.93 | 42.72 | 0 | 24.00 | 33.28 |
| S2-2 | 51.529 | 49.37 | 41.86 | 0 | 37.33 | 20.81 |
| S2-3 | 92.478 | 47.09 | 42.61 | 0 | 29.54 | 27.84 |
| S3-1 | 43.397 | 48.99 | 23.94 | 0 | 21.45 | 54.61 |
| S3-2 | 99.690 | 49.04 | 46.78 | 0 | 31.26 | 21.96 |
| S3-3 | 43.495 | 48.18 | 44.92 | 0 | 31.56 | 23.52 |
| R1-1 | 57.797 | 51.02 | 40.20 | 0 | 31.02 | 28.79 |
| R1-2 | 73.614 | 50.21 | 34.57 | 0 | 30.49 | 34.94 |
| R1-3 | 78.288 | 50.35 | 28.33 | 0 | 23.37 | 48.31 |
| R2-1 | 58.204 | 48.74 | 46.23 | 0 | 31.24 | 22.53 |
| R2-2 | 91.122 | 49.72 | 40.53 | 0 | 28.56 | 30.91 |
| R2-3 | 52.478 | 48.74 | 46.92 | 0 | 30.48 | 22.60 |
| R3-1 | 50.610 | 44.52 | 27.80 | 0 | 38.74 | 33.45 |
| R3-2 | 111.761 | 48.32 | 28.32 | 0 | 21.86 | 49.82 |
| R3-3 | 87.190 | 47.64 | 44.35 | 0 | 31.11 | 24.54 |

**Table S2.** Percentages of reads assigned to Bacteria, Archaea, Eukaryota and Viruses as well as not assigned reads obtained in Kraken2. S – susceptible plants, R – resistant plants, S/R(**1-3**) – number of biotype, S/R(1-3)-(**1-3**) – number of sample

| Sample | Reads | Classified [%] | Reads assigned to [%] | | | |
| --- | --- | --- | --- | --- | --- | --- |
|  |  |  | **Bacteria** | **Eukaryota** | **Archaea** | **Viruses** |
| S1-1 | 28898259 | 35.82 | 87.19 | 10.73 | 2.08 | 0.01 |
| S1-2 | 36806935 | 36.37 | 86.97 | 12.66 | 0.34 | 0.03 |
| S1-3 | 39144124 | 37.51 | 86.29 | 13.24 | 0.46 | 0.01 |
| S2-1 | 29101882 | 37.85 | 87.69 | 11.15 | 1.15 | 0.02 |
| S2-2 | 45561038 | 37.53 | 85.53 | 12.48 | 1.99 | 0.00 |
| S2-3 | 26238787 | 37.11 | 89.40 | 10.00 | 0.59 | 0.01 |
| S3-1 | 25304847 | 44.14 | 84.85 | 13.43 | 1.69 | 0.03 |
| S3-2 | 55880686 | 39.70 | 88.75 | 10.85 | 0.39 | 0.01 |
| S3-3 | 43595008 | 39.49 | 86.44 | 12.51 | 1.05 | 0.01 |
| R1-1 | 34569918 | 38.79 | 85.64 | 11.60 | 2.75 | 0.01 |
| R1-2 | 22765659 | 36.49 | 85.49 | 13.23 | 1.26 | 0.01 |
| R1-3 | 42380853 | 35.21 | 83.42 | 14.70 | 1.85 | 0.03 |
| R2-1 | 23885268 | 34.18 | 89.79 | 9.57 | 0.64 | 0.01 |
| R2-2 | 25764303 | 38.04 | 84.61 | 15.01 | 0.37 | 0.01 |
| R2-3 | 46238968 | 41.46 | 89.60 | 8.99 | 1.40 | 0.01 |
| R3-1 | 21698294 | 37.51 | 77.10 | 21.41 | 1.47 | 0.02 |
| R3-2 | 49845175 | 35.59 | 88.10 | 10.16 | 1.71 | 0.02 |
| R3-3 | 21747742 | 37.14 | 88.39 | 10.30 | 1.31 | 0.01 |

**Table S3.** Significantly differing reads abundancies of bacterial OTUs classified at family (**a**) and genus (**b**) level between two microbial communities derived from susceptible and resistant plants (p-value < 0.05, -2 ≥ fold change ≥ 2). Differential Abundance Analysis of Taxa performed between the abundances of reads assigned to individual OTUs identified in resistant *versus* sensitive to ALS inhibitors cornflower plants. Taxa presence in the analysed samples (out of 9 samples for each condition) is presented

| **Taxa Id** | **Scientific Name** | **Fold Change** | **p-value** | **Taxa presence in the analysed samples** | |
| --- | --- | --- | --- | --- | --- |
|  |  |  |  | **Susceptible** | **Resistant** |
| **(a) Family** | | | | | |
| 145358 | *Bogoriellaceae* | 2.6 | 0.014 | 9 | 9 |
| 1763524 | *Isosphaeraceae* | 2.2 | 0.029 | 9 | 9 |
| 186807 | *Peptococcaceae* | -2.0 | 0.002 | 9 | 9 |
| 1755824 | *Egicoccaceae* | -2.3 | 0.003 | 9 | 9 |
| 72275 | *Alteromonadaceae* | -2.3 | 0.026 | 9 | 9 |
| 2975441 | *Sphaerotilaceae* | -2.4 | 0.016 | 9 | 9 |
| 85025 | *Nocardiaceae* | -2.4 | 0.008 | 9 | 9 |
| 2008795 | *Azonexaceae* | -2.5 | 0.006 | 9 | 9 |
| 3018741 | *Cellulosilyticaceae* | -5.6 | 0.028 | 7 | 4 |
| 335929 | *Erythrobacteraceae* | -5.8 | 0.000 | 9 | 9 |
| **(b) Genus** | | | | | |
| 32207 | *Rothia* | 12.2 | 0.000 | 4 | 9 |
| 154116 | *Georgenia* | 2.8 | 0.011 | 9 | 9 |
| 943 | *Ehrlichia* | 2.2 | 0.041 | 9 | 9 |
| 1654931 | *Crenobacter* | 2.1 | 0.035 | 8 | 9 |
| 2651578 | *Thermanaerosceptrum* | -2.1 | 0.011 | 9 | 9 |
| 1755825 | *Egicoccus* | -2.2 | 0.004 | 9 | 9 |
| 626 | *Xenorhabdus* | -2.2 | 0.032 | 9 | 8 |
| 83461 | *Corallococcus* | -2.4 | 0.043 | 9 | 9 |
| 120831 | *Dyadobacter* | -2.4 | 0.002 | 9 | 9 |
| 1649463 | *Lentilitoribacter* | -2.5 | 0.048 | 9 | 9 |
| 165696 | *Novosphingobium* | -2.6 | 0.021 | 9 | 9 |
| 283735 | *Leeuwenhoekiella* | -3.4 | 0.044 | 9 | 4 |
| 511678 | *Aliivibrio* | -5.2 | 0.001 | 8 | 8 |
| 698776 | *Cellulosilyticum* | -5.4 | 0.032 | 7 | 4 |
| 1855416 | *Qipengyuania* | -6.9 | 0.000 | 9 | 9 |
| 1335745 | *Spiribacter* | -7.1 | 0.001 | 9 | 8 |

**References**

Burgos, N. R. *et al.* (2013) Confirmation of resistance to herbicides and evaluation of resistance levels. *Weed Sci.* **61**, 4-20.

European Herbicide Resistance Action Committee (2017) European guidelines to conduct herbicide resistance tests.

Marcinkowska, K. *et al.* (2023) Herbicidal ionic liquids containing double or triple anions as a new potential tool for weed control including herbicide-resistant biotypes. *Crop Protect.* **169**, 106238.

Panozzo, S., Scarabel, L., Collavo, A. & Sattin, M. (2015) Protocols for robust herbicide resistance testing in different weed species. *JoVE*, e52923.

Pernak, J., Niemczak, M., Rzemieniecki, T., Marcinkowska, K. & Praczyk, T. (2022) Dicationic herbicidal ionic liquids comprising two active ingredients exhibiting different modes of action. *J. Agric. Food Chem.* **70**, 2545-2553.

Stankiewicz-Kosyl, M. *et al.* (2021) Herbicide resistance of *Centaurea cyanus* L. in Poland in the context of its management. *Agronomy* **11**, 1954.
